# Which nocturnal hypoxemia parameters are associated with ambulatory blood pressure monitoring in adults with obstructive sleep apnea? A systematic review

**DOI:** 10.1007/s11325-026-03742-8

**Published:** 2026-07-03

**Authors:** Cíntia Felicio Adriano Rosa, Carolina Ferraz de Paula Soares, Michel Burihan Cahali

**Affiliations:** 1 Department of Otolaryngology. Faculdade de Medicina, Universidade de Sao Paulo, Sao Paulo, Brazil; 2 Hospital IPO, Curitiba, Brazil; 3 Medical School, Faculdade Assis Gurgacz, Cascavel, Brazil; 4 Department of Otolaryngology. Hospital das Clinicas HCFMUSP, Faculdade de Medicina, Universidade de Sao Paulo, Sao Paulo, Brazil

**Keywords:** Obstructive sleep apnea, Hypertension, Nocturnal hypoxemia, Oxygen desaturation index, Hypoxic burden

## Abstract

**Objective:**

Systemic arterial hypertension affects up to 50% of patients with obstructive sleep apnea (OSA) and represents one of the main modifiable cardiovascular risk factors. Hypoxemia is a recognized marker of OSA severity; however, it remains unclear which specific desaturation parameters during sleep, such as oxygen desaturation index (ODI), mean and minimum oxygen saturation (SpO_2_), percentage of sleep time with peripheral oxygen saturation < 90% (T90%) or hypoxic burden (HB) are most consistently associated with elevated blood pressure (BP). This systematic review aims to evaluate, in patients with OSA, the impact between nocturnal desaturation parameters and elevated BP as measured by ambulatory blood pressure monitoring (ABPM).

**Methods:**

A literature search was performed in PubMed, Web of Science, Scopus and Embase databases, covering all records available up to June 2025.

**Results:**

A total of 5,587 records were identified through database searches, and 24 studies met the inclusion criteria. The studies revealed heterogeneous results, with some showing significant associations between hypoxemia indices and hypertension, while others did not. Evidence suggests that ODI3% is commonly related to increased BP, whereas minimum SpO_2_, mean SpO_2_ and T90% yielded variable findings. HB was evaluated in only one study, suggesting a potential role in BP reduction after CPAP; however, the available evidence remains preliminary.

**Conclusion:**

ODI3% showed the most consistent association with increased BP across studies, whereas other hypoxemia indices yielded more variable results. Evidence regarding HB remains preliminary and warrants further investigation. These findings suggest that although ODI currently appears to be the most reliable marker, evidence from a single study indicates that composite indices such as HB may represent more informative predictors of BP outcomes and deserve further investigation in future studies.

## Introduction

Obstructive sleep apnea (OSA) is a respiratory disorder characterized by recurrent episodes of complete (apnea) or partial (hypopnea) collapse of the upper airway during sleep [1]. It is estimated to affect up to 23.4% of adult women and 49.7% of adult men [2]. Recent projections indicate that more than 1 billion individuals aged 30 to 69 years may be affected worldwide, with important clinical and public health implications [3]. Obstructive sleep apnea leads to periods of intermittent hypoxemia, sleep fragmentation, and repeated activation of the sympathetic nervous system, with significant pathophysiological consequences. Patients may experience excessive daytime sleepiness, reduced quality of life, and an increased risk of cardiovascular complications [4].

Hypertension is a prevalent condition that affects more than one-third of the global population and is strongly associated with OSA, particularly with the absence of the nocturnal blood pressure (BP) dip ("non-dipping") [5]. Systemic arterial hypertension affects up to 50% [6] of patients with OSA and represents one of the main modifiable risk factors for serious conditions such as myocardial infarction, stroke, and renal failure [5, 7]. The prevalence of OSA ranges from 64 to 83% among individuals with resistant hypertension [8, 9]. In this context, ambulatory blood pressure monitoring (ABPM) provides an accurate assessment of BP patterns over 24 h, making it a valuable tool to investigate the relationship between nocturnal hypoxemia and BP alterations in patients with OSA, as it minimizes the risk of missing a ‘true’ hypertension masked by nocturnal predominance [10].

There are some key mechanisms involved in the development of hypertension associated with OSA: repeated activation of carotid chemoreceptors with exaggerated reflex responses to hypoxemia; persistent sympathetic overactivity; and endothelial dysfunction [11, 12].Intermittent hypoxemia in OSA, characterized by cycles of hypoxia followed by reoxygenation, differs from sustained hypoxia and induces excessive production of reactive oxygen species, which may further contribute to the development of hypertension [12].

However, despite the recognition of hypoxemia as a marker of OSA severity, it remains unclear which specific desaturation parameters during sleep, such as oxygen desaturation index (ODI), mean and minimum oxygen saturation, percentage of sleep time with peripheral oxygen saturation (SpO_2_) < 90% (T90%) or hypoxic burden (HB) are most consistently associated with elevated BP.

This systematic review aims to identify, in patients with OSA, the impact between nocturnal desaturation parameters and elevated BP as measured by ABPM. This knowledge may contribute to the understanding of underlying pathophysiological mechanisms of hypertension in OSA and the identification of potential therapeutic response markers.

## Methods

The guiding research question for this review was: “In patients with OSA, which sleep-related hypoxemia markers, including ODI, mean and minimum SpO_2_ or T90% are independently associated with increased BP levels?’’ The research question was structured using the PICO acronym, as follows: Population: Adult patients with OSA; Intervention: Nocturnal hypoxemia parameters (HB, ODI, mean and minimum SpO_2_, T90%), Comparison: Absence or different levels of these parameters and Outcome: BP elevation/Hypertension.

The literature review was performed using the following medical databases: PubMed, Web of Science, Scopus and Embase. Health sciences descriptors (DeCS/MeSH terms) were used in the search strategy, with no restrictions regarding the fields of occurrence (e.g., title, abstract, keywords) or language. The descriptors used included: “Sleep Apnea, Obstructive’’ OR “Sleep Apnoea, Obstructive” OR ‘‘Apneas, Obstructive Sleep’’ OR ‘‘Obstructive Sleep Apnea’’ OR ‘‘Obstructive Sleep Apnea Syndrome’’ OR ‘‘Obstructive Sleep Apneas’’ OR ‘‘Sleep Apnea Hypopnea Syndrome’’ OR ‘‘Sleep Apnea Syndrome, Obstructive’’ OR ‘‘Sleep Apneas, Obstructive’’ OR ‘‘Syndrome, Obstructive Sleep Apnea’’ OR ‘‘Syndrome, Sleep Apnea, Obstructive’’ OR “OSA” OR “OSAS” AND "Hypoxic burden" OR "cumulative hypoxia" OR "hypoxia load" OR "hypoxic load" OR "oxygen desaturation burden" OR "hypoxemic load" OR "hypoxic exposure" OR "hypoxia severity" OR "intermittent hypoxia" OR "chronic intermittent hypoxia" OR "nocturnal hypoxemia" OR "oxygen desaturation index" OR "ODI" OR "ODI3" OR "ODI4" OR "desaturation index" OR "sleep oxygen desaturation" OR "nocturnal oxygen desaturation" OR "minimum SpO_2_" OR "lowest oxygen saturation" OR "oxygen saturation nadir" OR "nadir SpO2" OR "minimum oxygen saturation" OR "lowest nocturnal oxygen saturation" OR "SpO2 nadir" OR "average SpO_2_" OR "desaturation events" OR "nocturnal oxygen desaturation" OR "oxygen saturation below 90" OR "hypoxemia duration" OR "nocturnal hypoxemia" OR "sleep hypoxemia" OR "desaturation time" OR "desaturation severity" OR "hypoxia index" OR "T90" OR "time spent SpO2 < 90" OR "sleep time oxygen saturation < 90" AND "Hypertension" OR "High blood pressure" OR "Essential hypertension" OR "Arterial hypertension" OR "Blood pressure elevation" OR "Increased BP" OR "BP elevation" OR "elevated blood pressure" OR "essential hypertension" OR "HTN".

Studies were eligible if they included adult human participants (≥ 18 years) and presented original data from prospective, retrospective or cross-sectional observational studies, as well as randomized or non-randomized clinical trials. To be included, studies were required to: a) assess BP using ABPM, and b) report at least one nocturnal hypoxemia parameter, including ODI, minimum or mean SpO_2_, T90% or HB. There were no restrictions regarding publication date or language. Studies were excluded if they included only pediatric populations (< 18 years), involved animal models or were non-original publications, such as congress abstracts, letters to the editor, editorials, study protocols, comments or review articles (narrative or systematic). We predefined broad eligibility criteria to account for methodological diversity and variability in hypoxemia and ABPM assessment. Two investigators independently screened the titles and abstracts of all identified records to exclude studies that did not meet the eligibility criteria. Any disagreements were resolved by consensus. The following data were extracted from each included study: title, first author, year of publication, journal name, country of origin, study design, sample size, age and sex of participants, criteria for OSA diagnosis and severity classification, definition of hypopnea used, presence or absence of interventions (e.g., CPAP therapy, upper airway surgery), parameters of nocturnal hypoxemia (e.g., ODI, minimum and mean SpO_2_, T90%, HB), BP outcomes (e.g., 24-h BP, nighttime BP, dipping status), statistical analyses and main findings regarding the association between sleep-related hypoxemia and BP. The methodological quality of the included studies was appraised using the Joanna Briggs Institute (JBI) critical appraisal tools and the Cochrane Risk of Bias (RoB) tool, according to the specific design of each study. The JBI Critical Appraisal Checklist for Analytical Cross-Sectional Studies was applied to cross-sectional studies, and the JBI Checklist for Quasi-Experimental Studies (Before–After) was used for pre–post intervention studies [13, 14]. For randomized controlled trials (RCT), the risk of bias was assessed using the Cochrane Risk of Bias Tool [15]. All assessments were independently performed by two reviewers, and disagreements were resolved through discussion and consensus.

## Results

**Fig. 1:**
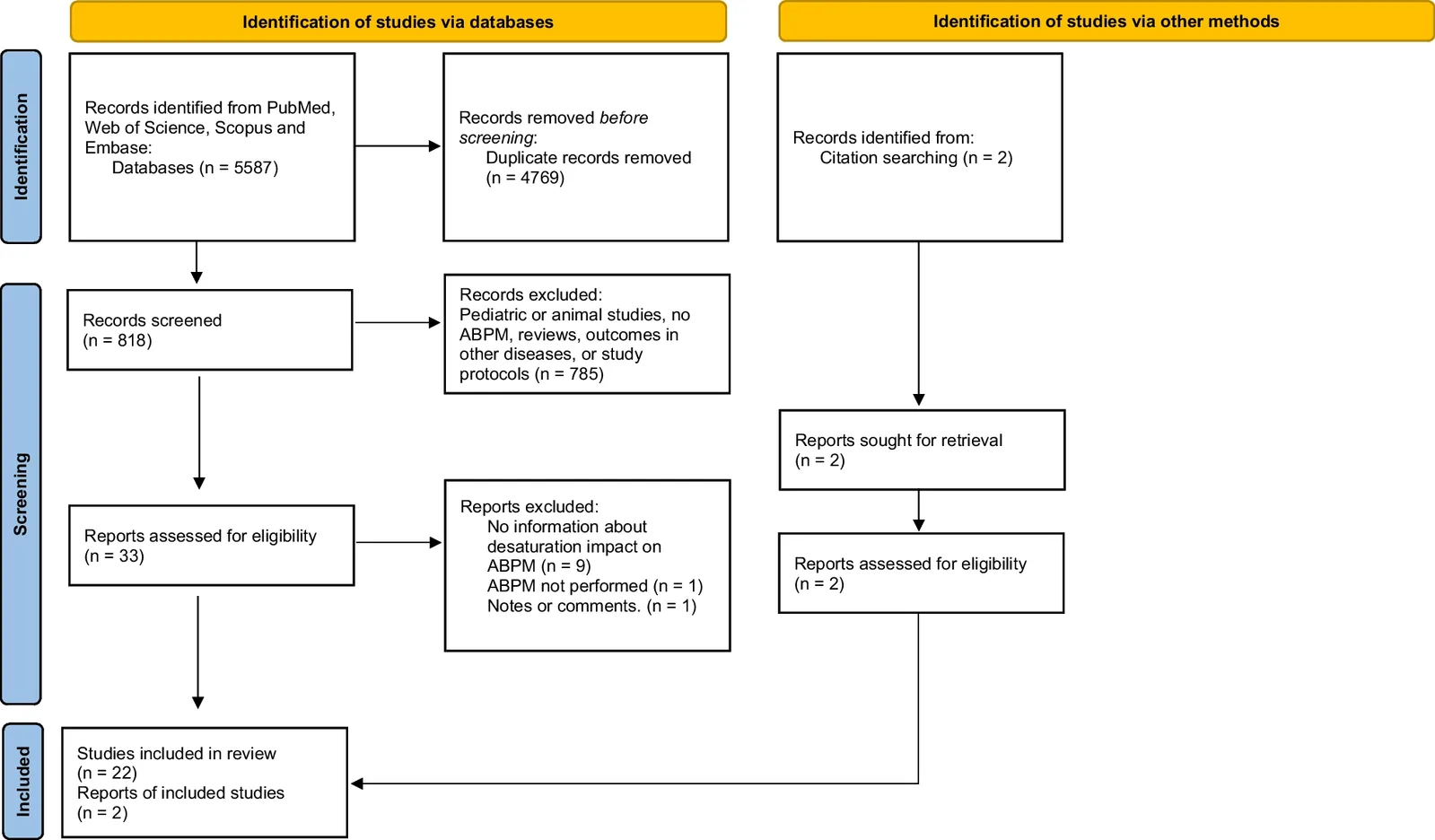
Flow diagram of study selection. Flow diagram of study selection. Figure adapted from the PRISMA 2020 flow diagram (http://www.prisma-statement.org/). Abbreviation: ABPM, ambulatory blood pressure monitoring

The database search was conducted up to June 23, 2025, and yielded a total of 5,587 records. After removing duplicates, 818 articles remained. Of these, 785 were excluded after title and abstract screening by the authors, leaving 33 articles for full-text review. Following full-text assessment, 22 studies met the eligibility criteria. In addition, the reference lists of the included articles were manually reviewed, and 2 additional studies were identified through hand-searching. In total, 24 studies were included in this review (Fig. 1).

Table 1 summarizes the data extracted from the studies included in this systematic review. In the text, for better comparability, the studies are grouped by the nocturnal hypoxemia markers evaluated and arranged chronologically. To further address clinical heterogeneity and provide a more refined phenotypic characterization, additional subgroup analyses were conducted according to hypertension severity and circadian blood pressure patterns. These findings are detailed in Tables 2, 3, and 4.

**Table 1:** Characteristics of the included studies assessing the relationship between ambulatory blood pressure monitoring and oxygen desaturation parameters during sleep

Author, year	Study type	Number of Patients	Study population and group characteristics	Intervention (Y/N and specify)	Hypoxemia parameters assessed	Main quantitative results
Suzuki et al., 1996 [25]	Analytical cross-sectional study	46	40 OSA (RDI ≥ 5) and 6 controls (RDI < 5)	N	Minimum SpO_2_; T90%	• Minimum SpO_2_ during sleep – no significant difference between dippers and non-dippers • T90% – no significant difference between dippers and non-dippers • Only significant predictor of non-dipping: RDI – systolic BP (*p* = 0.04), diastolic BP (*p* = 0.03)
Lo et al., 1998 [26]	Analytical cross-sectional study	54	OSA (*n* = 29) vs Controls (*n* = 25); 4 subgroups: OSA-HT (*n* = 14), OSA-NHT (*n* = 15), Control-HT (*n* = 10), Control-NHT (*n* = 15); OSA defined as AHI ≥ 5/h	N	Minimum SpO_2_	• Minimum SpO_2_ correlated with daytime SBP (*r* = –0.283, *p* < 0.05) and nighttime SBP (*r* = –0.419, *p* < 0.01)
García-Río et al., 2000 [27]	Analytical cross-sectional study	35	OSA defined as AHI ≥ 10/h and ≥ 70% obstructive apneas + daytime sleepiness. Four groups: Control (*n* = 11); Type 1 OSA – normotensive with nocturnal BP dipping (*n* = 10); Type 2 OSA – progressive BP rise overnight (*n* = 8); Type 3 OSA – HT without nocturnal BP dipping (*n* = 6)	N	Mean SpO_2_; Minimum SpO_2_	• Minimum SpO_2_ differed significantly in types 2 and 3 compared to the control group (control: 90 ± 2%; type 1: 81 ± 7%; type 2: 78 ± 7%; type 3: 78 ± 11%; *p* < 0.05) • Mean SpO_2_ values did not differ significantly between the subgroups and the control group
Liqiang et al., 2003 [35]	Analytical cross-sectional study	60	30 OSA with hypertension (OSA + HT) group and 30 pure OSA group	N	T90%	• OSA + HT group had significantly higher values of AHI, CAI, MAI, T90%, 24-h mean systolic BP (ASBP), and 24-h mean diastolic BP compared with the OSA group • Multiple linear regression analysis showed that, in the OSA + HT group, T90% was associated with the 24-h mean SBP and DBP (ASBP: *r* = 0.382, *P* < 0.05; ADBP: *r* = 0.369, *P* < 0.05)
Baguet et al., 2005 [28]	Analytical cross-sectional study	59	Patients with OSA (ODI ≥ 15/h): four groups – 25 HT and 34 non-HT by office BP; 45 HT and 14 non-HT by ABPM	N	Mean SpO_2_; Minimum SpO_2_	• No significant difference in SpO_2_ min between HT and NHT groups, either in the office setting (82 ± 9% vs. 82 ± 8%; *p* = 0.8) or on ABPM (82 ± 8% vs. 81 ± 7%; *p* = 0.7 • Mean nocturnal SpO_2_ was comparable between those with and without HT, whether defined by office BP measurement (93 ± 2% vs. 93 ± 2%; *p* = 0.6) or by ABPM (93 ± 2% vs. 93 ± 2%; *p* = 0.5)
Baguet et al., 2008 [29]	Analytical cross-sectional study	130	Three OSA groups (ODI ≥ 15/h): 41 normotensives; 39 masked HT – normal office BP, elevated ABPM; 46 HT – elevated office BP and ABPM	N	Mean SpO_2_; Minimum SpO_2_; T90%	• Minimum SpO_2_ (*p* = 0.97) did not differ significantly between the groups • Mean SpO_2_ was significantly lower in the hypertension and masked HT groups (93.8% ± 0.3 vs. 92.7% ± 0.5 vs. 93.0% ± 0.3; *p* = 0.04), despite no differences in RDI, minimum SpO_2_, or T90%
Grešová et al., 2008 [33]	Analytical cross-sectional study	116	Six groups: 15 healthy controls; 16 HT without OSA; 25 mild OSA (AHI ≥ 5 and ≤ 20/h); 23 moderate OSA (AHI > 20 and ≤ 40/h); 26 severe OSA (AHI > 40/h); 11 OSA on CPAP	CPAP therapy in 11 patients	Mean SpO_2_; T90%; T85%	• Mean SpO_2_ was negatively correlated with nighttime DBP (*r* = −0.279; *p* = 0.0457) • T85% was correlated with nighttime DBP (*r* = 0.287; *p* = 0.049) and 24-h DBP (*r* = 0.326; *p* = 0.0104)
Navarro- Antolín et al., 2009 [30]	Prospective non-randomized pre–post CPAP study	20	Men aged 35–65 years, OSA (mostly severe)	3-month CPAP therapy	Minimum SpO_2_	• CPAP corrected nocturnal hypoxemia, reducing SBP/DBP by 6.5/5.3 mmHg • Minimum SpO_2_ (correlated PAMS 24 h *r*^2^ = 0.52; *p* < 0.05)
Drager et al., 2010 [31]	Comparative cross-sectional case–control study	61	Two groups: 43 OSA and 18 controls (AHI < 5/h)/Two groups: 13 OSA with masked HT (MH) and 30 OSA without MH. OSA: AHI ≥ 15/h, type 1 PSG	N	Minimum SpO_2_; T90%	• Minimum SpO_2_ was lower in OSA with MH vs without MH (70% ± 12 vs 78% ± 7; *p* = 0.04). In univariate analysis, minimum SpO_2_ (*r* = –0.54, *p* < 0.001) and AHI (*r* = 0.37, *p* = 0.003) correlated with arterial stiffness. In multivariate regression, only minimum SpO_2_ (*β* = –0.051, *p* = 0.02) and daytime SBP (*β* = 0.058, *p* = 0.005) remained independently associated with increased arterial stiffness
Assoumou et al., 2012 [16]	Prospective population-based cohort study	806	Elderly cohort (mean age 68 ± 0.8 years) from the PROOF study: two groups – with MetS (*n* = 79) and without MetS (*n* = 727); subgroups – OSA without MetS (*n* = 393) and OSA with MetS (*n* = 56). OSA: AHI > 15/h with ≥ 85% obstructive events	N	ODI3%, Mean SpO_2_; Minimum SpO_2_; T90%	• ODI3% was associated with MetS (*p* < 0.0001) and independently correlated with DBP in OSA patients (*r* = 0.180, *p* < 0.01), but not with SBP • Mean SpO_2_ also did not differ significantly between those with and without MetS (95.3 ± 0.07 vs. 94.6 ± 0.3; *p* = 0.06) • Minimum SpO_2_ (88.7 ± 0.2 vs. 87.1 ± 0.6) was not significantly associated with the presence of MetS among elderly patients with OSA (AHI > 15/h) • T90% was associated with MetS in elderly OSA patients (*p* < 0.0001)
Roche et al., 2012 [17]	Analytical cross-sectional observational study using data from the PROOF–SYNAPSE cohort	470	Patients (mean age 68 years) untreated for HT and without prior OSA diagnosis: classified by OSA severity – AHI < 15 (*n* = 234); AHI 15–30 (*n* = 155); AHI > 30 (*n* = 81)	N	ODI3%	• ODI3% > 10/h was the parameter most strongly associated with increased BP. After adjustment, associated with higher odds of systolic hypertension (OR = 4.94, 95% CI: 2.10–11.74, *p* = 0.0003) and diastolic hypertension (OR = 4.31, 95% CI: 2.08–8.92, *p* = 0.0003) in elderly OSA patients. Association with Masked HT
Martinez-Garcia et al., 2013 [36]	Multicenter randomized controlled trial	194	OSA (AHI ≥ 15/h) with resistant hypertension: two groups – CPAP group (*n* = 98) and control group (*n* = 96)	3-month CPAP therapy	T90%	• After adjustment, T90% was the only variable associated with all BP response outcomes: 24-h SBP ↓5 mmHg (OR = 1.06, 95% CI: 1.02–1.10, *p* = 0.014); 24-h DBP ↓4 mmHg (OR = 1.04, 95% CI: 1.02–1.30, *p* = 0.022); nocturnal SBP ↓4 mmHg (OR = 1.06, 95% CI: 1.01–1.10, *p* = 0.022); nocturnal DBP ↓2 mmHg (OR = 1.04, 95% CI: 1.00–1.10, *p* = 0.045); recovery of dipper pattern (OR = 1.06, 95% CI: 1.03–1.10, *p* = 0.036). Severe OSA (AHI ≥ 30/h) was associated only with greater nocturnal BP reductions (SBP –6.7 mmHg, 95% CI: –1.4 to –10.8; DBP –3.7 mmHg, 95% CI: –0.9 to –7.1) and recovery of dipper pattern
Soares et al., 2014 [37]	Prospective before-and-after study in patients undergoing surgery	18	Patients with OSA > 5/h or primary snoring, with or without HT (mostly normotensive)	Lateral pharyngoplasty – 6 months	T90%	• After 6 months, AHI, arousals, and T90% decreased significantly, but changes were not correlated with BP improvements
Sova et al., 2014 [18]	Analytical cross-sectional study	97	AHI > 15, indicated for CPAP. Masked HT defined as normal office BP but elevated ABPM in any period. Nocturnal hypertension defined as nighttime BP > 120/70 mmHg regardless of office BP	N	ODI; Mean SpO_2_; T90%	• ODI – higher in patients with nocturnal HT (*p* = 0.002); no significant correlation with masked HT (prevalence 56.7%) • Mean SpO_2_ – no significant correlation with masked HT (*p* = 0.991) or nocturnal HT (*p* = 0.167) • T90% – no significant correlation with masked HT (*p* = 0.896) or nocturnal HT (*p* = 0.516)
Wei et al., 2015 [19]	Retrospective analytical comparative observational study	151	Three groups: 56 men with OSA and HT; 50 age-, BMI-, and neck circumference–matched controls with isolated OSA; 45 men with isolated HT	Some patients used APAP	ODI, Mean SpO_2_; Minimum SpO_2_; T90%	• ODI – higher in OSA + HT vs pure OSA (56.86 vs 39.42; *p* < 0.05); correlated with ΔSBP (*r* = 0.212, *p* = 0.011) and ΔDBP (*r* = 0.218, *p* = 0.031) after CPAP treatment • Mean SpO_2_ – lower in OSA + HT vs pure OSA vs controls (74.77 ± 8.04 vs 83.08 ± 6.65 vs 95.08 ± 2.45; *p* < 0.05); correlated with SBP (*r* = –0.202, *p* = 0.039) and DBP (*r* = –0.244, *p* = 0.042) • Minimum SpO_2_ – lower in OSA + HT vs pure OSA vs controls (66.02 ± 8.75 vs 72.08 ± 11.42 vs 90.8 ± 2.42; *p* < 0.05); correlated with SBP (*r* = –0.310, *p* = 0.032) and DBP (*r* = –0.291, *p* = 0.023) • T90% – no association with SBP (*r* = 0.067, *p* = 0.711) or DBP (*r* = 0.069, *p* = 0.781)
Casitas et al., 2017 [32]	Double-blind randomized controlled trial	31	Isolated nocturnal HT group (*n* = 15) and sustained HT group (*n* = 16). All patients with AHI ≥ 10/h	CPAP vs sham	Mean SpO_2_; Minimum SpO_2_	• No baseline differences in AHI, Mean SpO_2_ or SpO_2_ min between isolated nocturnal or sustained HT; no correlation analysis between hypoxemia parameters and BP performed
Guimarães et al., 2018 [20]	Analytical cross-sectional study	120	Two groups: 35 controls (AHI < 5/h, arousal index < 15/h, ESS < 10) and 85 mild OSA (AHI > 5/h and ≤ 15/h) regardless of excessive daytime sleepiness. Sub-analysis – four groups by OSA and nocturnal BP dipping: 6 controls non-dippers, 29 controls dippers, 29 mild OSA non-dippers, and 56 mild OSA dippers	N	Mean SpO_2_; Minimum SpO_2_, ODI4; T90%	• ODI4% – higher in mild OSA dippers vs non-dippers (*p* < 0,05); male sex, age, and the nondipping pattern were associated with the augmentation index (vascular stiffness), independently of ODI4%, in both groups of individuals with AHI < 15 • SpO_2_ min, Mean SpO_2_ and T90% – no difference between mild OSA dippers and non-dippers
Warchol-Celinska et al., 2018 [21]	Open-label phase II randomized controlled trial without placebo/sham group	60	Two groups: 30 resistant HT patients undergoing renal denervation and 30 resistant HT patients on medication. All with moderate-to-severe OSA	Renal denervation – 3- and 6-month follow-up; CPAP in 8 RD and 7 control patients	ODI3; T90%; Minimum SpO_2_; Mean SpO_2_	• ODI – ↓ in RD group (38.3 ± 27.9 → 30.7 ± 25.3; *p* = 0.018); no change in control group (32.5 ± 21.7 → 29.2 ± 29.2; *p* = 0.16); between RD resistant HT group vs. resistant HT control group NS (*p* = 0.29). ↓ in RD (*p* = 0.018); • Minimum SpO_2_– no significant difference • Mean SpO_2_—significant between-group difference (*p* = 0.028) • T90% – no significant difference
Sapiña-Beltran et al., 2019 [22]	Cross-sectional association study ancillary to a larger cohort	284	Patients with resistant HT and OSA: *n* = 237; control group without OSA: *n* = 47	N	ODI4; Minimum SpO_2_; Mean SpO; T90%	• ODI4, Minimum SpO_2_ and Mean SpO_2_ – no significant difference between controlled and uncontrolled resistant HT groups • T90% – similar in controlled vs uncontrolled resistant HT (24-h ABPM)
Thomas et al., 2021 [34]	Analytical cross-sectional observational study	130	Two groups: refractory HT (*n* = 37) and controlled resistant HT (*n* = 93). OSA severity categorized as: none (AHI < 5/h), mild (AHI ≥ 5/h and < 15/h), moderate (AHI ≥ 15/h and < 30/h), and severe (AHI ≥ 30/h)	22 on CPAP	Mean SpO_2_; T90%	• Mean SpO_2_ and T90% – no significant difference between refractory and controlled resistant HT patients
Lloberes et al., 2022 [38]	Analytical cross-sectional observational study	142	OSA > 20/h: divided into two groups by nocturia episodes – < 2 episodes (*n* = 92) and ≥ 2 episodes (*n* = 50)	N	T90%	• T90% – independently associated with nocturnal HT (OR = 1.02, 95% CI: 1–1.04, *p* = 0.043); cut-off ≥ 35% predicted outcome. Combination with nocturia increased likelihood of nocturnal HT • T90%—no significant difference between Dipper vs. non-dipper (*p* = 0.078)
Seah et al., 2023 [23]	Analytical cross-sectional observational study	106	Adults > 40 years with HT and OSA (AHI ≥ 15/h)	N	Hourly variability of ODI3, and minimum SpO_2_	• Hour-to-hour variability in ODI3% – associated with 24-h pulse pressure (*p* = 0.0018), 24-h SBP (*p* = 0.0151), and daytime SBP (*p* = 0.0061); remained independently associated with 24-h pulse pressure after adjustment (*β* = 0.450, 95% CI: 0.174–0.726, *p* = 0.002) • Minimum SpO_2_ – no association with 24-h pulse pressure (*p* = 0.8604), 24-h SBP (*p* = 0.6605), or waking SBP (*p* = 0.8727), unlike AHI and ODI
Messineo et al., 2024 [39]	Prospective cohort study with secondary analysis of an RCT	168	AHI 15–50: divided into CPAP group (*n* = 87) and supplemental O_2_ group (*n* = 81)	CPAP vs supplemental O_2_ 2 L/min	HB	• HB – treatment-related HB reduction associated with BP reduction. For every 2 standards deviation (SD) reduction in HB, ↓SBP (–4.2 mmHg, 95% CI: 0.9–7.5, *p* = 0.013) and ↓MAP (–2.5 mmHg, 95% CI: 0.2–4.8, *p* = 0.035), not DBP. Greatest BP reductions in highest HB reduction tertile (Q3) vs Q1, more pronounced in CPAP group; no association for AHI or ODI • Dippers with HBchange in Q3 vs. Q1—reduction in SBP (6.7 [0.7; 12.6] mmHg, *P* = 0.020
Tangutur et al., 2024 [24]	Prospective before-and-after study in patients undergoing surgery	41	Patients with OSA ≥ 5/h and indication for upper airway surgery	OSA surgery (various types) – 6 months	ODI; T90%	• ODI and T90% – no correlation with BP changes after 6 months, no significant reductions in 24-h, daytime, or nighttime BP

Table 1 Description of study design, sample characteristics, methods and main outcomes related to ABPM and desaturation

Abbreviations: *ABPM* ambulatory blood pressure monitoring; *AHI* apnea–hypopnea index; *APAP* auto-adjusting positive airway pressure; *BMI* body mass index; *BP* blood pressure; *CAI* central apnea index; *CPAP* continuous positive airway pressure; *DBP* diastolic BP; *ΔDBP* change in diastolic BP; *ΔSBP* change in systolic BP; *ESS* Epworth Sleepiness Scale; *HB* hypoxic burden; *HT* hypertension; *MAI* mixed apnea index; *MAP* mean arterial pressure; *MetS* metabolic syndrome; *NHT* non-hypertension; *NS* non-significant; *OSA* obstructive sleep apnea; *ODI* oxygen desaturation index; *RDI* respiratory disturbance index; *RD* renal denervation; *SBP* systolic BP; *SpO*_2_, peripheral oxygen saturation; *T90%*, time with SpO_2_ < 90%

**Table 2:** Hypoxemia Parameters and Dipper vs Non-dipper Pattern

Author, year	OSA severity (study-level) */Treatment	Dipper vs Non-dipper pattern (ABPM)	Hypoxemia parameters assessed
Suzuki et al., 1996 [25]	Severe	Dipper vs. non-dipper	• Minimum SpO_2_—no significant difference between dipper vs. non-dipper • T90%—no significant difference between dipper vs. non-dipper
García-Río et al., 2000 [27]	Severe	Dipper vs. non-dipper	• Minimum SpO_2_—differed significantly between control and non-dipper • Mean SpO_2_- no significant difference between subgroups or controls
Martinez-Garcia et al., 2013 [36]	Severe **(3-month CPAP therapy)**	Dipper vs. non-dipper	• After adjustment, T90% was associated with recovery of dipper pattern
Guimarães et al., 2018 [20]	Mild	Dipper vs. non-dipper	• ODI4% – higher in dippers with mild OSA • Minimum SpO_2_, Mean SpO_2_and T90% – no difference between mild OSA dippers and non-dippers
Lloberes et al., 2022 [38]	Severe	Dipper vs. non-dipper	• T90%—no significant difference between dipper vs. non-dipper
Messineo et al., 2024 [39]	Moderate **3-month CPAP vs supplemental O**_2_** 2 L/min**	Dipper vs. non-dipper	• HB – non-dippers showed less BP benefit from CPAP compared with dippers

Table 2 Description of hypoxemia parameters and dipper vs non-dipper pattern

Abbreviations: *ABPM* ambulatory blood pressure monitoring; *BP* blood pressure; *CPAP* continuous positive airway pressure; *HB* hypoxic burden; *OSA* obstructive sleep apnea; *ODI* oxygen desaturation index; *SpO*_2_, peripheral oxygen saturation; *T90%* time with SpO_2_ < 90%. *Based on group mean AHI

**Table 3:** Hypoxemia Parameters and associated Nocturnal BP

Author, year	OSA severity (study-level) */Treatment	Nocturnal BP (ABPM)	Hypoxemia parameters assessed
Lo et al., 1998 [26]	Severe	Nocturnal BP	• Minimum SpO_2_—correlated with nocturnal SBP (*r* = –0.419, *p* < 0.01)
Grešová et al., 2008 [33]	3 OSA severity groups **11 on CPAP**	Nocturnal BP	• Mean SpO_2_—negatively correlated with nocturnal DBP (*r* = −0.279; *p* = 0.0457) • T85%—correlated with nocturnal DB (*r* = 0.287; *p* = 0.049)
Martinez-Garcia et al., 2013 [36]	Severe **(3-month CPAP therapy)**	Nocturnal BP	• T90%—associated with BP reduction after CPAP in resistant HT: 24-h SBP ↓5 mmHg (OR = 1.06, 95% CI: 1.02–1.10, *p* = 0.014); 24-h DBP ↓4 mmHg (OR = 1.04, 95% CI: 1.02–1.30, *p* = 0.022); nocturnal SBP ↓4 mmHg (OR = 1.06, 95% CI: 1.01–1.10, *p* = 0.022); nocturnal DBP ↓2 mmHg (OR = 1.04, 95% CI: 1.00–1.10, *p* = 0.045);
Soares et al., 2014 [37]	Severe **Lateral pharyngoplasty – 6 months**	Nocturnal BP	• T90%—not correlated with nocturnal DBP and SBP reductions
Sova et al., 2014 [18]	Severe	Nocturnal BP	• ODI – higher in patients with nocturnal HT (*p* = 0.002) • Mean SpO_2_ – no significant correlation with nocturnal HT • T90% – no significant correlation with nocturnal HT
Casitas et al., 2017 [32]	Severe **CPAP vs sham (2 × 12 weeks)**	Nocturnal BP	• Mean SpO_2_ – no difference between isolated nocturnal or sustained HT • Minimum SpO_2_– no difference between isolated nocturnal or sustained HT
Lloberes et al., 2022 [38]	Severe	Nocturnal BP	• T90% – independently associated with nocturnal HT (OR = 1.02, 95% CI: 1–1.04, *p* = 0.043); cut-off ≥ 35% predicted outcome. Combination with nocturia increased likelihood of nocturnal HT
Messineo et al., 2024 [39]	Moderate **3-month CPAP vs supplemental O**_2_** 2 L/min**	Nocturnal BP	• HB – treatment-related HB reduction associated with nocturnal SBP reduction (4.6 mmHg per 2SD increase in HB change, 95% CI:1.2- 8.0, *P* = 0.009)
Tangutur et al., 2024 [24]	Severe **OSA surgery (various types) – 6 months**	Nocturnal BP	• ODI and T90% – no correlation with BP changes after 6 months, no significant reductions in 24-h, daytime, or nocturnal BP

Table 3 Description of hypoxemia parameters and associated Nocturnal hypertension

Abbreviations: *ABPM* ambulatory blood pressure monitoring; *BP* blood pressure; *CPAP* continuous positive airway pressure; *DBP* diastolic BP; *HB* hypoxic burden; *HT* hypertension; *OSA* obstructive sleep apnea; *ODI* oxygen desaturation index; *SBP* systolic BP; *SpO*_2_, peripheral oxygen saturation; *T90%,* time with SpO_2_ < 90%. *Based on group mean AHI

**Table 4:** Hypoxemia Parameters and associated Hypertension Phenotypes

Author, year	OSA severity (study-level) */Treatment	Hypertension phenotype	Hypoxemia parameters assessed
Baguet et al., 2008 [29]	Severe	Masked HTN	• Mean SpO_2_—lower in HT and masked HT vs normotensive • Minimum SpO_2_—no significant difference between groups • T90%—no significant difference between groups
Drager et al., 2010 [31]	Severe	Masked HTN	• Minimum SpO_2_—lower in OSA with masked HT vs without masked HT • T90%—no significant difference between groups
Roche et al., 2012 [17]	Most participants had mild to moderate OSA **Elderly OSA patients**	Masked HTN	• ODI3% > 10/h—association with masked HT
Sova et al., 2014 [18]	Severe	Masked HTN	• ODI – no significant correlation with masked HT • Mean SpO_2_ – no significant correlation with masked HT • T90% – no significant correlation with masked HT
Martinez-Garcia et al., 2013 [36]	Severe **(3-month CPAP therapy)**	Resistant HT	• T90%—associated with BP reduction after CPAP in resistant HT: 24-h SBP ↓5 mmHg; 24-h DBP ↓4 mmHg; nocturnal SBP ↓4 mmHg; nocturnal DBP ↓2 mmHg
Warchol-Celinska et al., 2018 [21]	Severe **Renal denervation (RD) – 3 and 6-month follow-up; CPAP in 8 RD**	Resistant HT	• ODI – ↓ in RD (*p* = 0.018); no significant difference between RD resistant HT group vs. resistant HT control group (*p* = 0.29) • Minimum SpO_2_ – no significant difference • Mean SpO_2_—significant between-group difference (*p* = 0.028) • T90% – no significant difference
Sapiña-Beltran et al., 2019 [22]	Moderate	Resistant HT	• ODI4, Minimum SpO_2_ and Mean SpO_2_ – no significant difference between controlled vs. uncontrolled resistant HT groups • T90%—no significant difference between controlled vs. uncontrolled resistant HT after adjusting for age, sex, and BMI
Thomas et al., 2021 [34]	Mild Effective AHI calculated in 22 **CPAP users**	Resistant HT vs Refractory HT	• Mean SpO_2_ and T90% – no significant difference between refractory HT vs. controlled resistant HT patients

Table 4 Description of hypoxemia parameters and associated Hypertension phenotypes

Abbreviations: *ABPM* ambulatory blood pressure monitoring; *AHI* apnea–hypopnea index; *BMI* body mass index; *BP* blood pressure; *CPAP* continuous positive airway pressure; *DBP* diastolic BP; *ΔDBP* change in diastolic BP; *ΔSBP* change in systolic BP; *HT* hypertension; *MAP* mean arterial pressure; *OSA* obstructive sleep apnea; *ODI* oxygen desaturation index; *RD* renal denervation; *SBP* systolic BP; *SpO*_2_ peripheral oxygen saturation; *T90%* time with SpO_2_ < 90%. *Based on group mean AHI

**Fig. 2:**
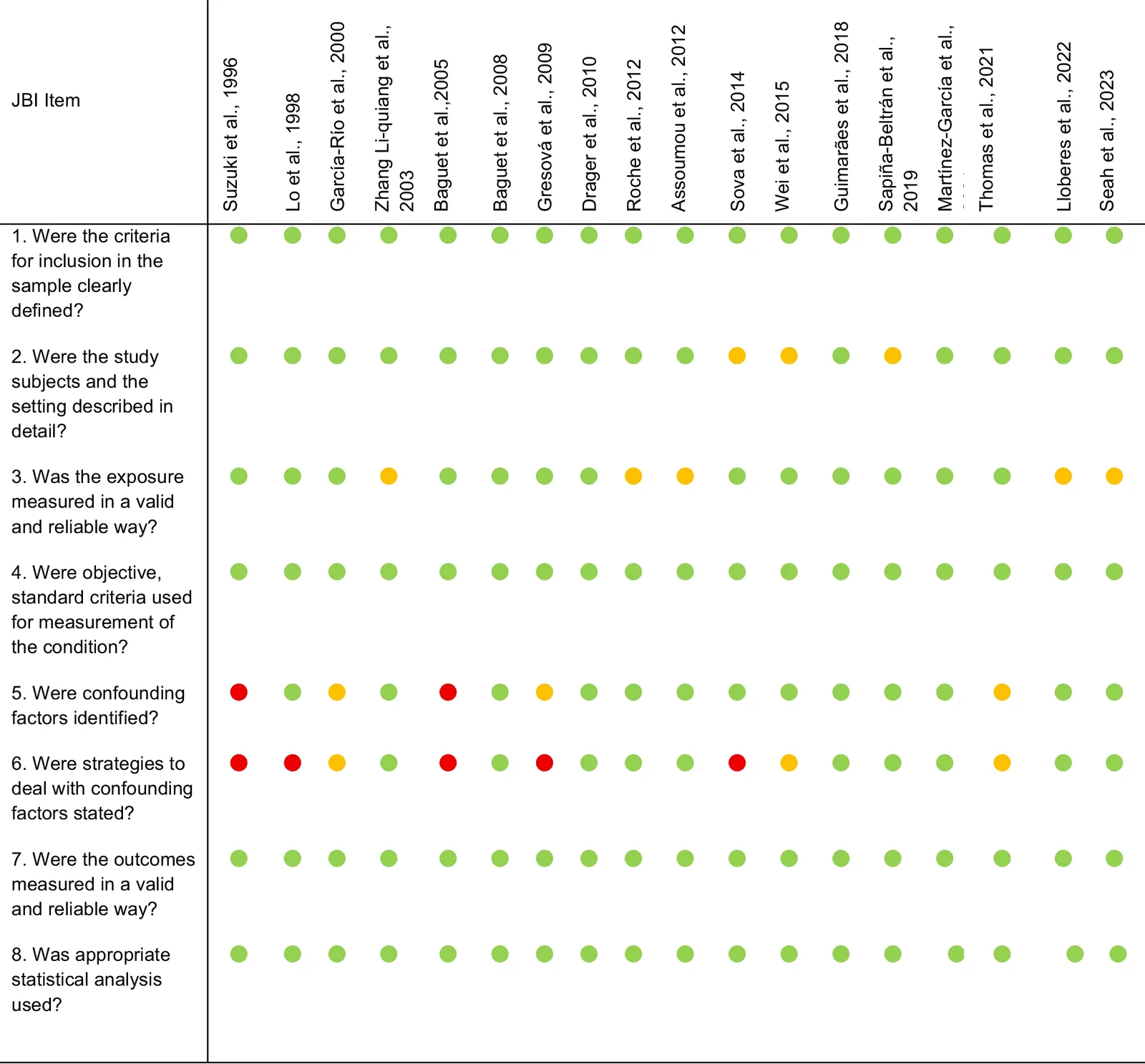
Quality appraisal using Joanna Briggs institute tool Cross-Sectional Studies by study. Legend: Green = Yes; Yellow = Unclear; Red = No. Adapted from the JBI Critical Appraisal Checklist for Analytical Cross-Sectional Studies (JBI, 2020) [13]

**Fig. 3:**
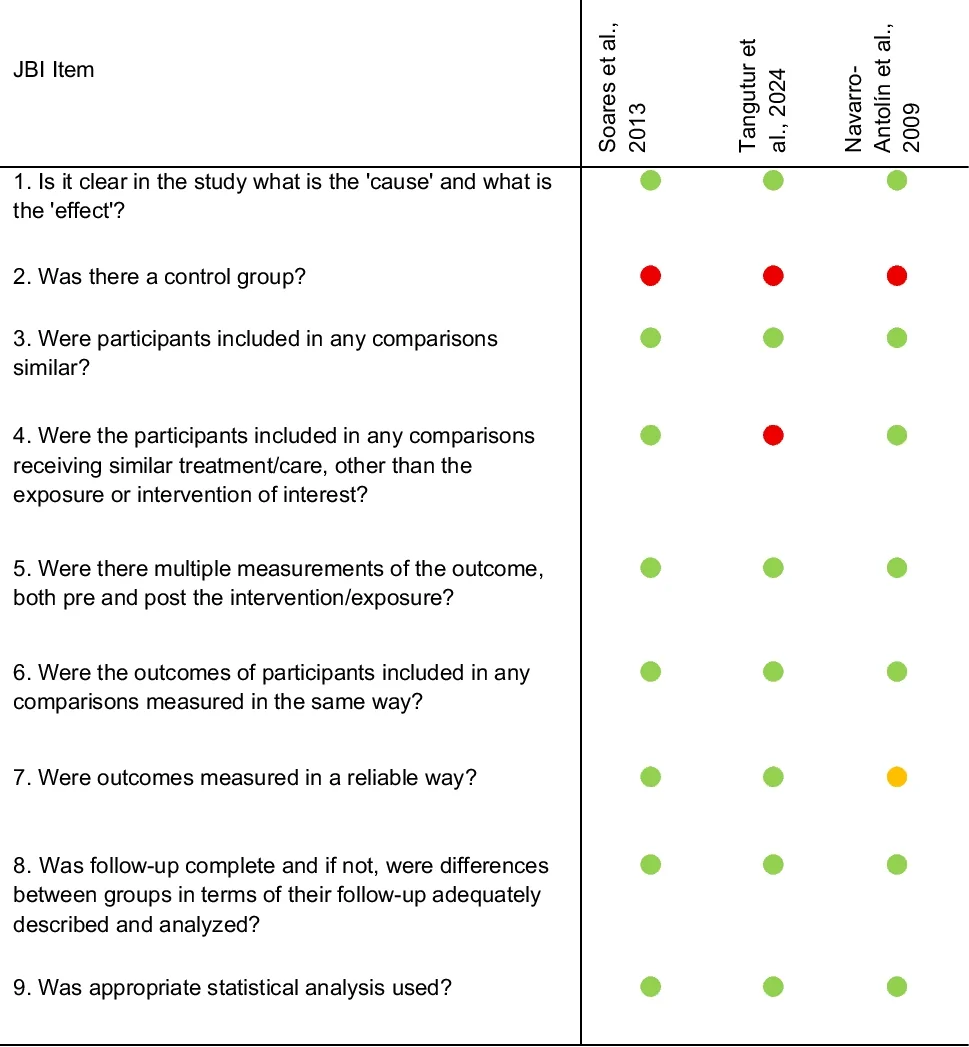
Quality appraisal using Joanna Briggs institute tool Quasi Experimental Study (Before–After) by study. Legend: Green = Yes; Yellow = Unclear; Red = No. Adapted from the JBI Checklist for Quasi-Experimental Studies (Before–After) [14]

**Fig. 4:**
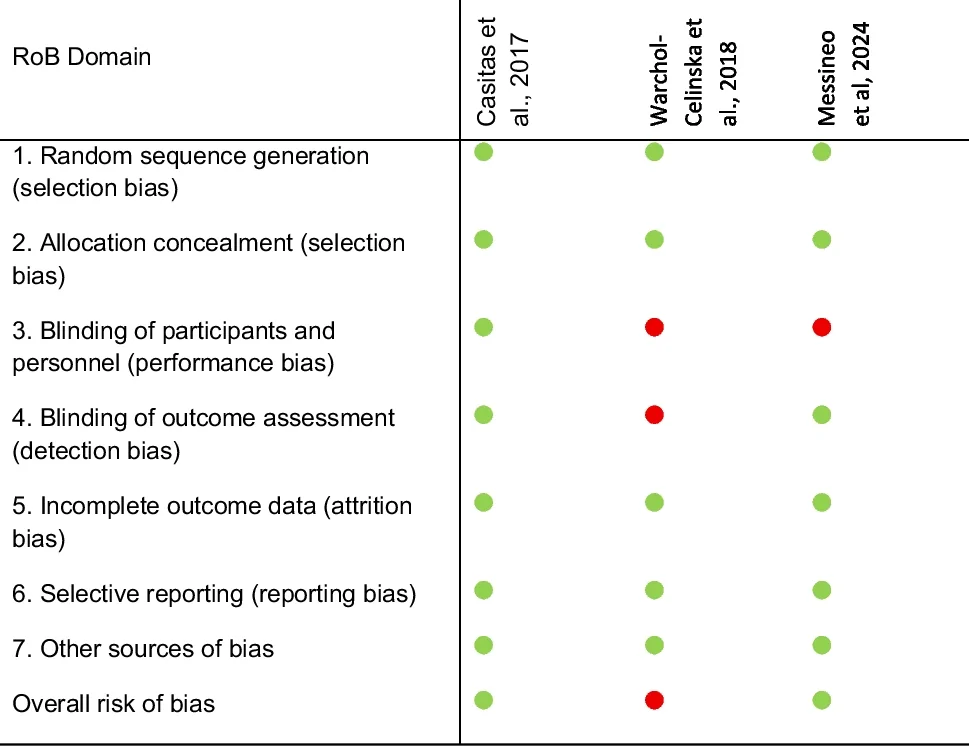
Risk of bias assessment of randomized controlled trials using the Cochrane RoB tool (Higgins et al., 2011). Legend: Green = low risk, Red = high risk, Yellow = insufficient information. Adapted from the Cochrane Risk of Bias Tool (Higgins et al., 2011) [15]

The overall methodological quality of the studies ranged from moderate to high according to the criteria of the applied instruments. Specifically, among the cross-sectional studies, the mean score on the JBI Critical Appraisal Checklist for Analytical Cross-Sectional Studies ranged from 6 to 8 “Yes” responses (mean = 6.8). The most frequently fulfilled domains were those related to clearly defined inclusion criteria and the use of valid and reliable measures for both exposure and outcomes, whereas the identification and control of potential confounding factors represented the most common limitation. Similarly, in the pre–post studies assessed using the JBI Checklist for Quasi-Experimental Studies, most reports presented clear descriptions of participants and appropriate statistical analyses; however, none included a control group. Regarding the RCTs, evaluated with the Cochrane Risk of Bias (RoB) tool, most showed a low risk of selection and performance bias. As expected for some interventions, blinding of participants was not feasible, although the outcomes were objectively assessed. Figures 2, 3, and 4 summarize the individual quality assessments according to the applied instruments.

The key findings are presented below.

### Oxygen desaturation index

Oxygen Desaturation Index was assessed in 9 of the included studies. In a prospective cohort of 806 elderly individuals (mean age 68 ± 0.8 years) from the PROOF study, Assoumou et al. (2012) found that ODI3% was associated with the presence of metabolic syndrome (MetS) (*p* < 0.0001) and showed an independent correlation with diastolic blood pressure (DBP) among patients with OSA (*r* = 0.180; *p* < 0.01), but not with systolic blood pressure (SBP) [16]. Roche et al. (2012) collected data from 470 patients in the PROOF-SYNAPSE cohort in a cross-sectional observational study and found that, in elderly individuals, an ODI3% > 10/h was the parameter strongly associated with increased BP. After adjustment for confounding variables, ODI3% was associated with a higher likelihood of systolic hypertension (OR = 4.94; 95% CI: 2.10–11.74; *p* = 0.0003) and diastolic hypertension (OR = 4.31; 95% CI: 2.08–8.92; *p* = 0.0003) in elderly patients with OSA [17]. In a cross-sectional study of 97 patients with OSA (AHI > 15/h), Sova et al. (2014) reported significantly higher ODI values in those with nocturnal hypertension (*p* = 0.002). The prevalence of masked hypertension was high (56.7%); however, no significant correlation was found between ODI and masked hypertension [18]. Wei et al. (2015), in a retrospective study of 151 patients, divided participants into three groups: OSA with hypertension (HT), OSA without HT, and HT without OSA. The OSA with HT group showed higher ODI values than the pure OSA group (56.86 vs. 39.42; *p* < 0.05). In addition, ODI was significantly correlated with change from baseline in systolic blood pressure (ΔSBP) and change from baseline in diastolic blood pressure (ΔDBP) values after CPAP treatment (r: 0,212; *p* = 0,011 and r: 0,218; *p* = 0,031 respectively) [19]. In 2018, Guimarães et al. studied 85 patients with mild OSA and 35 controls, subdividing them according to the presence of nocturnal dipping, and assessed peripheral vascular endothelial function using Endo-PAT. Unexpectedly, ODI4% was higher in the mild OSA dipping group compared to the nondipping group. Male sex, age, and the nondipping pattern were associated with the augmentation index (vascular stiffness), independently of ODI4%, in both groups of individuals with AHI < 15 [20]. Warchol-Celinska et al. (2018), in a non-blinded, non-placebo-controlled RCT with 30 patients with moderate-to-severe OSA undergoing renal denervation and 30 patients with resistant hypertension, some of whom were using CPAP, observed a significant reduction in both systolic and diastolic BP, during both daytime and nighttime, in the renal denervation group. At the 3-month follow-up, the RDN group showed a significant reduction in ODI3% (38.3 ± 27.9 to 30.7 ± 25.3; *p* = 0.018), whereas no significant change was observed in the control group (32.5 ± 21.7 to 29.2 ± 29.2; *p* = 0.16) [21]. However, the between-group difference in ODI3% change did not reach statistical significance (*p* = 0.29). In a cross-sectional study including 284 patients with resistant hypertension, Sapiña-Beltrán et al. (2019) analyzed both controlled and uncontrolled cases and compared patients with OSA (*n* = 237) to controls without OSA (*n* = 47). The authors reported a high prevalence of OSA (83.5%) and a dose–response relationship between OSA severity (AHI) and BP values; nonetheless, ODI4% did not differ between controlled and uncontrolled resistant hypertension groups as defined by 24-h ambulatory BP monitoring [22]. Seah et al. (2023), in an interesting cross-sectional study involving 106 patients with OSA ≥ 15/h, observed that hour-to-hour variability in ODI3% was associated with 24-h pulse pressure (*p* = 0.0018), 24-h SBP (*p* = 0.0151), and daytime SBP (*p* = 0.0061). After adjusting for potential confounders including age, sex, body mass index, history of stroke, mean apnea hypopnea index (AHI)/ODI, number of hour-to-hour PSG recordings, and percentage of REM sleep, hour-to-hour variability in ODI3% remained independently associated with 24-h pulse pressure (β coefficient, 0.450 [95% CI = 0.174–0.726], *p* = 0.002) [23]. In a prospective study, involving 41 patients with OSA ≥ 5/h who underwent various types of OSA surgery, using an intraindividual paired design, Tangutur et al. (2024) found no correlation between changes in ODI4% and BP after 6 months. Moreover, no statistically significant reductions were observed in 24-h, daytime, or nighttime BP [24].

### Minimum oxygen saturation

Regarding minimum SpO_2_, 13 studies investigated its association with BP outcomes. In 1996, Suzuki et al. (1996), in an observational study with 46 patients: 40 with OSA (RDI ≥ 5) and 6 controls (RDI < 5), found that the lowest oxygen saturation during sleep did not differ significantly between dippers and non-dippers [25]. Lo et al., in 1998, in a cross-sectional study, evaluated 54 patients, mostly men, divided into groups according to the presence of OSA (AHI ≥ 5/h) and hypertension. There were no significant differences between the groups in age, sex, or BMI. The minimum SpO_2_ correlated with daytime systolic mean arterial pressure (*r* = –0.283, *P* < 0.05) and nighttime systolic mean arterial pressure (*r* = –0.419, *P* < 0.01). Greater sympathetic activation was observed, as evidenced by elevated urinary catecholamine levels and an increase in heart rate proportional to the decrease in SpO_2_ and the duration of apneas [26]. García-Río et al. (2000), in a cross-sectional study with 35 patients, divided into groups according to the presence of OSA (AHI ≥ 10/h and a percentage of obstructive apneas ≥ 70%, combined with daytime hypersomnolence) and BP levels: Group 1 – OSA with normal BP (SBP < 140 mmHg and DBP < 90 mmHg) over 24 h and with nocturnal dipping present; Group 2 – OSA with a progressive increase in BP from the onset of sleep until early morning; Group 3 – hypertensive OSA with elevated BP (systolic ≥ 140 mmHg and/or diastolic ≥ 90 mmHg) over 24 h, without nocturnal dipping; and a control group. The minimum SpO_2_ differed significantly between types 2 and 3 compared to the control group (control: 90 ± 2%; type 1: 81 ± 7%; type 2: 78 ± 7%; type 3: 78 ± 11%; *p* < 0.05) [27]. This finding suggests that, in patients with OSA, particularly those exhibiting progressive nocturnal BP elevation or non-dipping hypertension, nocturnal hypoxemia is more pronounced compared to controls. This association indicates that greater severity of oxygen desaturation during sleep may contribute to abnormal circadian BP patterns, reinforcing the potential role of hypoxemia in the disruption of nocturnal blood pressure regulation*.* Baguet et al. (2005), in a cross-sectional study with 59 patients with OSA (RDI ≥ 15/h), compared according to the presence of hypertension as determined by office blood pressure measurement and by ABPM, found no significant difference in minimum SpO_2_ between hypertensive and normotensive groups, either in the office setting (82 ± 9% vs. 82 ± 8%; *p* = 0.8) or on ABPM (82 ± 8% vs. 81 ± 7%; *p* = 0.7) [28]. Similarly, in another study by the same author (2008), 130 patients with OSA (RDI ≥ 15/h) were divided into groups: normotensive, masked hypertension, and hypertension (elevated BP in both office and ABPM). The minimum SpO_2_ (*p* = 0.97) did not differ significantly between the groups [29]. A prospective, non-randomized clinical study (2009) with a molecular approach compared pre- and post-treatment with CPAP over 3 months in 20 men aged 35 to 65 years, most with severe OSA, combining ABMP, nocturnal hypoxemia assessment, and gene expression analysis of the Maxi-K⁺ channel β1-subunit in peripheral leukocytes. Correction of nocturnal hypoxemia with CPAP use resulted in significant reduction in systolic and diastolic BP by 6.5 mmHg and 5.3 mmHg, respectively. The more severe the nocturnal hypoxemia (as measured by minimum SpO_2_), the lower the expression of the Maxi-K⁺ channel β1-subunit in leukocytes [30]. Drager et al. (2010), in a cross-sectional case–control study with 61 patients divided into groups: 43 with OSA (AHI ≥ 15/h) and 18 controls (AHI < 5/h), and further stratified into 13 OSA patients with masked hypertension (MH) and 30 OSA patients without MH, found that the minimum SpO_2_ was significantly lower in the OSA with MH group than in those without MH (70% ± 12 vs. 78% ± 7; *p* = 0.04). In addition, minimum SpO_2_ and AHI correlated with arterial stiffness in univariate analysis (*r* = –0.54; *p* < 0.001 and *r* = 0.37; *p* = 0.003, respectively). In the multivariate regression model, only minimum SpO_2_ and daytime SBP remained independently associated with increased arterial stiffness (*β* = –0.051; *p* = 0.02 and *β* = 0.058; *p* = 0.005, respectively) [31]. In the study by Wei et al. (2015) mentioned above, the OSA with hypertension group showed lower minimum SpO_2_ values than the pure OSA group and controls (66.02% ± 8.75 vs. 72.08% ± 11.42 vs. 90.8% ± 2.42; *p* < 0.05). Similar to the ODI, minimum SpO_2_ was significantly correlated with SBP and DBP (*r* = –0.310; *p* = 0.032 and *r* = –0.291; *p* = 0.023, respectively) [19]. Casitas et al. (2017), in a double-blind RCT of patients with OSA (AHI ≥ 10/h) assigned to CPAP or sham treatment, included 31 patients with isolated or sustained nocturnal hypertension. Despite finding no differences between groups in baseline AHI or minimum SpO_2_, no direct correlation analysis was performed between hypoxemia data and BP values [32]. In the study by Guimarães et al. (2018) involving 85 patients with mild OSA and 35 controls, with an analysis of dipping patterns, no difference in minimum SpO_2_ was found between the mild OSA dipper and non-dipper groups [20]. In the study by Warchol-Celinska et al. (2018), evaluating the renal denervation, minimum SpO_2_ improved in both groups; however, the between-group difference was not statistically significant [21]. Sapiña-Beltrán et al. (2019) reported no significant differences in minimum SpO_2_ between controlled and uncontrolled resistant hypertension groups [22]. In the study by Seah et al. (2023), which evaluated the hour-to-hour variability of sleep respiratory indices, minimum SpO_2_ was not associated with 24-h pulse pressure (*p* = 0.8604), 24-h SBP (*p* = 0.6605), or waking SBP (*p* = 0.8727), in contrast to AHI and ODI [23].

### Mean oxygen saturation

Concerning mean SpO_2_ values during sleep, 12 studies were reviewed. In the study by García-Río et al. (2000), involving 35 patients stratified by OSA status and nocturnal BP pattern, mean SpO_2_ values did not differ significantly among BP groups [27]. Also, in the study by Baguet et al. (2005), mean nocturnal SpO_2_ was comparable between those with and without hypertension, whether defined by office BP measurement (93 ± 2% vs. 93 ± 2%; *p* = 0.6) or by ABPM (93 ± 2% vs. 93 ± 2%; *p* = 0.5) [28]. In contrast, Baguet et al. (2008), found that mean SpO_2_ was significantly lower in the hypertension and masked hypertension groups (93.8% ± 0.3 vs. 92.7% ± 0.5 vs. 93.0% ± 0.3; *p* = 0.04), despite no differences in respiratory disturbance index (RDI), minimum SpO_2_, or T90% [29]. It is worth noting that no direct correlation analyses were performed between hypoxemia parameters and BP levels obtained from ABPM. In a cross-sectional observational study, Grešová et al. (2008) evaluated 116 participants divided into six groups: healthy controls (*n* = 15), patients with hypertension without OSA (*n* = 16), mild OSA (AHI ≥ 5 and ≤ 20/h; *n* = 25), moderate OSA (AHI > 20 and ≤ 40/h; *n* = 23), severe OSA (AHI > 40/h; *n* = 26), and OSA patients on CPAP therapy (*n* = 11). Mean SpO_2_ was negatively correlated with nighttime DBP (*r* = 0.279; *p* = 0.0457) [33]. In the cross-sectional study by Sova et al. (2014), mean SpO_2_ showed no significant correlation with masked hypertension (*p* = 0.991) or nocturnal hypertension (*p* = 0.167) [15]. Wei et al. (2015) found that the OSA with hypertension group had lower mean SpO_2_ values than non-hypertensive OSA group and controls (74.77% ± 8.04 vs. 83.08% ± 6.65 vs. 95.08% ± 2.45; *p* < 0.05). Similar to the ODI and minimum SpO_2_, mean SpO_2_ was significantly correlated with SBP and DBP (*r* = –0.202; *p* = 0.039 and *r* = –0.244; *p* = 0.042, respectively) [19]. As previously mentioned for minimum SpO_2_, Casitas et al. (2017) also found no differences in mean SpO_2_ between patients with isolated or sustained nocturnal hypertension assigned to CPAP or sham treatment, but no direct correlation analysis was performed between hypoxemia parameters and BP values [32]. Guimarães et al. (2018) reported that mean SpO_2_ was comparable between the mild OSA dipper and non-dipper groups2^0^. In the RCT by Warchol et al. (2018), the renal denervation group showed a reduction in AHI (39.4 ± 25.5 to 31.2 ± 23.4; –8.1 [–14.6 to –1.7]; *p* = 0.015) and an improvement in mean SpO_2_ (%) (92.1 ± 4.2 to 93.5 ± 2.1; 1.4 [–0.5 to 2.0]; *p* = 0.057), with a statistically significant between-group difference, unlike T90% and minimum SpO_2_ [21]. In contrast, Sapiña-Beltrán et al. (2019) also found no significant differences in mean SpO_2_ between the controlled and uncontrolled resistant hypertension groups [22]. Thomas et al. (2021), in a cross-sectional study of 130 patients, compared two groups: refractory hypertensive (*n* = 37) and controlled resistant hypertensive (*n* = 93), of whom 22 were using CPAP. Nocturnal hypoxemia did not differ between the groups, with no significant differences in AHI or mean nocturnal SpO_2_ [34].

### Time with SpO_2_ below 90%

With respect to T90%, defined as the proportion of total sleep time with SpO_2_ < 90%, 14 of the included studies reported on this parameter. Suzuki et al. observed that T90% did not differ significantly between dippers and non-dippers [25]. The only significant predictor of a non-dipping pattern was RDI, both for SBP (*p* = 0.04) and DBP (*p* = 0.03). All participants with an RDI ≤ 5 exhibited the expected physiological BP decline, whether normotensive or hypertensive. Liqiang et al. (2003), in a cross-sectional study with 60 patients with OSA divided according to the presence of hypertension, found that the OSA + HT group had significantly higher values of AHI, central apnea index (CAI), mixed apnea index (MAI), T90%, compared with the OSA group. Moreover, in the OSA + HT group, T90% was associated with the 24-h mean SBP and DBP (ASBP: *r* = 0.382, *P* < 0.05; ADBP: *r* = 0.369, *P* < 0.05) [35]. The duration of SpO_2_ < 85% (T85%) was correlated with nighttime DBP (*r* = 0.287; *p* = 0.049) and 24-h DBP (*r* = 0.326; *p* = 0.0104) in a cross-sectional observational study (Gresova et al. 2008) involving 116 patients divided into groups according to healthy control status, presence of hypertension, and OSA severity, with some patients using CPAP [33]. In the study by Baguet et al. (2008), T90% (*p* = 0.38), similar to minimum SpO_2_, did not differ significantly among normotensive and hypertensive OSA patients [29]. Drager et al. (2010) revealed that there were no significant differences in AHI or T90% among controls and OSA with or without masked hypertension groups, despite the SpO_2_ min being lower in OSA patients with MH [31]. Martínez-García et al. (2013), in a multicenter RCT conducted in Spain with 194 patients with OSA (AHI ≥ 15/h) and resistant hypertension, randomized participants into a CPAP group (*n* = 98) and a control group (*n* = 96) for 12 weeks. After adjustment for confounding factors, T90% was the only variable associated with all BP response outcomes: 24-h SBP reduction of 5 mmHg: T90% OR 1.06 (95% CI: 1.02–1.10; *p* = 0.014); 24-h DBP reduction of 4 mmHg: T90% OR 1.04 (95% CI: 1.02–1.30; *p* = 0.022); nocturnal SBP reduction of 4 mmHg: T90% OR 1.06 (95% CI: 1.01–1.10; *p* = 0.022); nocturnal DBP reduction of 2 mmHg: T90% OR 1.04 (95% CI: 1.00–1.10; *p* = 0.045); and recovery of the dipper pattern: T90% OR 1.06 (95% CI: 1.03–1.10; *p* = 0.036). The presence of severe OSA in the CPAP group (AHI ≥ 30/h) was associated with greater reductions in nocturnal BP (–6.7 mmHg [95% CI: –1.4 to –10.8] for SBP and –3.7 mmHg [95% CI: –0.9 to –7.1] for DBP), as well as recovery of the dipper pattern. Thus, the authors identified a clinical phenotype of better responders to CPAP treatment, represented by patients with both higher T90% values and severe OSA [36]. In the study by Soares et al. (2014), 18 patients with primary snoring or OSA (AHI > 5/h), mostly (83%) normotensive in ABPM, underwent lateral pharyngoplasty. After 6 months, patients showed significant reductions in AHI, arousals, T90%, and in ABPM. However, neither T90% nor other polysomnographic variables correlated with improvements in BP [37]. Similar to the findings with mean SpO_2_, Sova et al. (2014), noted that T90% showed no significant correlation with masked hypertension (*p* = 0.896) or nocturnal hypertension (*p* = 0.516) [18]. In the study by Wei et al. (2015), which demonstrated significant associations for ODI, minimum, and mean SpO_2_, no association was found for T90% with SBP (*r* = 0.067; *p* = 0.711) or DBP (*r* = 0.069; *p* = 0.781) [19]. As previously noted for minimum SpO_2_, Warchol-Celinska et al. (2018) also found no statistically significant difference in T90% between the renal denervation and control groups, despite overall improvements in BP parameters [21]. As with mean SpO_2_, Guimarães et al. (2018) found no significant difference in T90% between mild OSA patients classified as dippers and those classified as non-dippers [20]. Similarly, Sapiña-Beltrán et al. (2019) found similar T90% values in patients with controlled and uncontrolled resistant hypertension [22]. In the study by Thomas et al. (2021), T90% did not differ significantly between refractory and controlled resistant hypertensive patients [34]. In contrast, Lloberes et al. (2022), in a cross-sectional study of 142 patients with OSA (AHI > 20/h), found that T90% was independently associated with nocturnal hypertension (OR = 1.02; 95% CI: 1–1.04; *p* = 0.043), with a cut-off ≥ 35% predicting this outcome. Although nocturia was not an independent predictor, its combination with elevated T90% increased the likelihood of nocturnal hypertension [38]. As previously described for ODI, the prospective study (2024) of 41 patients with OSA (AHI ≥ 5/h) undergoing various types of OSA surgery also found no correlation between T90% and changes in BP after 6 months [24].

### Hypoxic burden

The only study identified that specifically evaluated HB in relation to BP outcomes was conducted by Messineo et al. (2024), as a prospective cohort with a secondary analysis of an RCT [39]. This study evaluated 168 patients with OSA (15 ≤ AHI ≤ 50/h) randomized to CPAP (*n* = 87) or supplemental oxygen at 2 L/min (*n* = 81). Treatment-related reductions in HB were significantly associated with decreases in SBP and mean arterial pressure (MAP), but not DBP. For every 2 standard deviation (SD) reduction in HB, SBP decreased by an estimated 4.2 mmHg (95% CI: 0.9 to 7.5; *p* = 0.013) and MAP by 2.5 mmHg (95% CI: 0.2 to 4.8; *p* = 0.035), whereas the change in DBP was not statistically significant (–1.9 mmHg; *p* = 0.066). Exploratory analyses by tertiles of HB change showed that patients in the highest tertile (Q3) had substantially greater reductions in SBP (–6.5 mmHg; *p* = 0.001), DBP (–2.6 mmHg; *p* = 0.031), and MAP (–3.52 mmHg; *p* = 0.011) compared to those in the lowest tertile (Q1). These reductions were observed in both nocturnal SBP (–4.6 mmHg; *p* = 0.009) and daytime SBP (–4.1 mmHg; *p* = 0.029), with the most pronounced effect in the CPAP group. Among “dippers” with high HB reduction (Q3), SBP decreased by 6.7 mmHg compared with Q1 (*p* = 0.020). No association was observed between reductions in AHI or ODI and decreases in BP, and in the control group, HB change was not significantly related to BP improvement [39].

## Discussion

Our systematic review analyzed the relationship between different markers of nocturnal hypoxemia and BP in patients with OSA. Overall, the studies revealed heterogeneous results, with some showing significant associations between hypoxemia indices and hypertension, while others did not. This variability underscores the complexity of the pathophysiological links between intermittent hypoxemia and BP regulation and highlights the importance of carefully interpreting which markers best capture the cardiovascular burden of OSA.

### Oxygen desaturation index

Findings across studies remain heterogeneous. Six investigations demonstrated an association between higher ODI and increased BP on ABPM [16–19, 21, 23], whereas five did not [16, 18, 20, 22, 24]. Importantly, some reports yielded divergent results within their own analyses, suggesting that the relationship between ODI and BP may vary according to the parameter assessed (systolic vs. diastolic, daytime vs. nighttime) and the methodological approach employed [16, 18]. For example, ODI was associated with nocturnal hypertension but not with masked hypertension [18], indicating that desaturation may contribute directly to nocturnal BP elevation, whereas masked hypertension is likely influenced by additional factors independent of hypoxemia [40]. Although an ODI > 10 events/h was associated with higher BP in the PROOF-SYNAPSE cohort [17], most included studies analyzed ODI as a continuous variable, which precluded the establishment of a universal severity cutoff. Discrepancies may also relate to inclusion criteria: in normotensive individuals by office measurements (and therefore not receiving antihypertensive therapy), ODI was more clearly associated with both SBP and DBP [17], whereas in patients with established hypertension, associations were restricted to DBP [16]. This pattern align with the concept that DBP, more closely linked to peripheral vascular resistance, may be sensitive to intermittent hypoxemia, whereas SBP mainly reflects long-term arterial stiffness [41, 42]. Another observation was that studies using ODI3% tended to show stronger correlations with BP parameters than those using ODI4%, likely reflecting the higher sensitivity of the 3% threshold in detecting milder desaturation events with cardiovascular relevance, particularly in mild to moderate OSA. However, these findings should be interpreted with caution, as no study directly compared the two desaturation thresholds within the same population, and some reports did not specify the SpO_2_ drop criterion used to calculate ODI [18, 19]. Nevertheless, evidence also supports ODI4% as a predictor of hypertension: in a large study without ABPM, ODI4% was independently associated with hypertension [43], whereas ODI3% ≥ 30/h predicted office hypertension in men [44]. Notably, unlike the cumulative indices used in most studies, one investigation evaluated the hour-to-hour variability of ODI3% and demonstrated significant associations with 24-h and daytime SBP [23]. While this approach limits comparability, it raises the intriguing hypothesis that the temporal distribution of desaturations may be relevant for BP regulation. Methodological heterogeneity in desaturation definitions therefore contributes to inconsistent results across studies. Overall, higher ODI values, particularly ODI3%, are frequently associated with increased BP parameters, although associations vary according to population characteristics, methodological choices, and the specific BP outcome assessed.

### Minimum oxygen saturation

Evidence from 13 studies evaluating minimum SpO_2_ and BP outcomes in OSA is inconsistent. Five investigations found lower values in patients with masked, non-dipping, or resistant hypertension and reported correlations with higher nighttime BP, greater arterial stiffness, or even molecular alterations reversible with CPAP [19, 26, 27, 30, 31]. However, eight studies did not detect differences across groups [20–23, 25, 28, 29, 32], even when distinct OSA severities or hypertension phenotypes were considered. Taken together, these discrepancies likely reflect the limitations of minimum SpO_2_ as an isolated index, since it represents only the single lowest desaturation value, which may be affected by artifacts or isolated events.

### Mean oxygen saturation

The association between mean SpO_2_ and BP outcomes in OSA has been inconsistent across 12 studies. Some investigations reported lower mean SpO_2_ in subgroups with hypertension or masked hypertension [19, 29], modest correlations with systolic and diastolic BP [19, 33] and improvement following renal sympathetic denervation, with a significant between-group difference [18]. In contrast, most studies, including those comparing office versus ABPM-defined hypertension, controlled versus uncontrolled resistant hypertension, or different dipping patterns, found no significant differences or consistent associations [18, 20–22, 28, 32, 34]. These inconsistencies likely reflects the limitations of mean SpO_2_ as an index, since it represents only an arithmetic average of the entire night, which dilutes the variability between periods of hypoxemia and normoxemia. As such, it does not distinguish individuals with intense intermittent hypoxemia, characterized by repeated desaturation peaks but relatively preserved average values, from those with more prolonged and sustained desaturations. Moreover, mean SpO_2_ may also capture desaturation episodes unrelated to sleep apnea, such as technical artifacts, arousals, or respiratory comorbidities, further reducing its specificity as a cardiovascular marker. These limitations may obscure the intermittent hypoxemia that drives sympathetic activation and vascular dysfunction. These findings suggest that mean SpO_2_, in isolation, may play a limited role as a discriminatory marker of hypertension in patients with OSA.

### Time with SpO_2_ below 90%

Results from 14 studies evaluating T90% and BP outcomes in OSA were heterogeneous. Three investigations identified T90% as an independent predictor of nocturnal hypertension or of BP response to CPAP, and higher T90% values were associated with increased 24-h SBP and DBP in hypertensive patients [35, 36, 38]. Interestingly, even when using a slightly different threshold, T85% was also correlated with nighttime and 24-h DBP, further supporting the potential relevance of cumulative hypoxemia indices for BP regulation [33]. However, 11 studies reported no significant differences between groups or correlations with BP parameters [18–22, 24, 25, 29, 31, 34, 37].

Beyond the studies included in our review, additional studies have further explored the prognostic significance of T90%. Tan et al., 2022 with meta-analyses have shown that T90% is associated with all-cancer mortality and only the severe group (> 12%) compared with none or mild (< 2.1%) showed an OR of 2.66 (1.21–5.85) [45]. Moderate and severe desaturation (defined by T90%) among patients with OSA was associated with a 30–40% higher risk of all-cancer [45]. Martínez et al. proposed T90% > 8% as an additional parameter, and Labarca et al. concluded, after unadjusted analysis, that T90% > 20% and minimum SpO_2_ < 75% were associated with risk of hypertension, type 2 diabetes, greater EDS, and mortality [46, 47]. These data reinforce the potential clinical value of T90%, particularly when appropriate thresholds are applied, but its role as a predictor of BP outcomes in OSA remains inconsistent across smaller studies, highlighting the need for more robust cutoff points to define its association with hypertension.T90% reflects not only the depth but also the cumulative duration of hypoxemia, which may be more closely linked to vascular remodeling, oxidative stress, and chronic sympathetic activation. Nevertheless, T90% may fail to capture the intermittency of events and it be influenced by diseases not related to sleep apnea which reduces its specificity as a marker of hypertension in this population. Methodological differences across studies also help explain the heterogeneity of results. Based on these data, T90% appears to have a limited and conflicting role as a marker of HT in patients with OSA; however, when an association was observed, it was independently related to BP parameters and, according to a single study, may predict a better response to CPAP therapy.

### Hypoxic burden

Unlike the AHI, which only counts the number of apnea and hypopnea events per hour, HB considers the depth (amplitude) and duration of oxygen desaturation in each respiratory event. Recent studies have shown that HB has stronger correlations with cardiovascular outcomes and mortality than AHI, and it is considered a sensitive and clinically relevant marker of OSA severity [48–51]. A recent review suggested that an HB value above 60%min/h could be considered a threshold to identify OSA cases at higher risk of cardiovascular morbidity and mortality [49]. Although only one study (Messineo et al., 2024) in this present review examined HB, this index proved to be a more relevant predictor of BP response to CPAP treatment than conventional indices such as AHI or ODI, although further research is needed to confirm this association. Treatment-related reductions in HB were significantly associated with decreases in SBP and MAP, but not DBP [39]. The exclusion criteria (AHI > 50 or minimum SpO_2_ < 85%) narrowed the spectrum of OSA severity and reduced the mean HB in the sample, potentially limiting the generalizability of the findings.

Some studies defined nocturnal hypertension based on absolute nighttime BP thresholds (e.g., > 120/70 mmHg), whereas others relied on the absence of nocturnal dipping (< 10% decline). These methodological differences may partly explain the variability in reported associations between nocturnal hypoxemia indices and BP outcomes. Two studies evaluated minimum SpO_2_ and nocturnal BP, with inconsistent findings: one reported a correlation with nocturnal SBP [26], whereas the other did not [32]. Regarding mean SpO_2_, one study reported a negative correlation with nocturnal DBP [33], whereas two others found no significant correlation with nocturnal hypertension or no differences between isolated nocturnal and sustained hypertension [18, 32]. Five studies evaluated T90% (or T85%) in relation to nocturnal BP. One study reported that T90% was independently associated with nocturnal hypertension, with a cut-off ≥ 35% predicting the outcome and the combination with nocturia increasing the likelihood of nocturnal hypertension [38]. Two studies found no significant association between T90% and nocturnal hypertension or nocturnal BP changes. One study reported that T85% correlated with nocturnal DBP [33]. In resistant hypertension, another study found that T90% was associated with BP reduction after CPAP therapy, including reductions in nocturnal SBP and DBP [36]. Two studies evaluated ODI in relation to nocturnal BP. One reported higher ODI in patients with nocturnal hypertension [18], whereas the other found no correlation with BP changes 6 months after OSA surgery (various types) and no significant reductions in nocturnal BP [24]. Only one study evaluated hypoxic burden and reported that CPAP treatment–related reductions in HB were associated with reductions in nocturnal SBP [39].

In subgroup analyses based on dipper pattern, results were inconsistent. Three studies evaluated the association between the dipper pattern and minimum SpO_2_ [20, 25, 27]. Only one reported a significant difference reporting lower minimum SpO_2_ in non-dippers compared with dippers [27]. Mean SpO_2_ was assessed in two studies, neither of which found significant differences between groups [20, 27]. One study reported that ODI4% was higher in dippers with mild OSA compared with non-dippers [20]. T90% was not significantly associated with the dipper pattern in four studies [20, 25, 36, 38]; however, one interventional study using CPAP reported its association with recovery of the dipper pattern [36]. HB was evaluated in only one study, and an exploratory analysis suggested that non-dippers experienced less BP reduction with CPAP compared with dippers [39].

Current evidence does not support a consistent association between hypoxemia metrics and hypertension severity stages like masked and resistant hypertension phenotypes. Two studies investigated minimum SpO_2_ and masked hypertension with conflicting results [29, 31]. Similar inconsistent findings were reported for mean SpO_2_ [18, 29]; and three studies reported no significant differences in T90% [18, 29, 31]. Regarding resistant hypertension, one study found no significant differences in ODI4%, SpO_2_ min, mean SpO_2_, or T90% [22]. Another reported that T90% was associated with BP reduction after CPAP therapy, including reductions in 24-h SBP and DBP, nocturnal SBP and DBP [36]. In a study of resistant hypertension undergoing renal denervation, ODI decreased after the procedure but did not differ from the control group; mean SpO_2_ showed a significant between-group difference, whereas SpO_2_ min and T90% showed no significant differences [21]. Mean SpO_2_ and T90% did not differ significantly between patients with refractory hypertension and controlled resistant hypertension [34].

Another important source of heterogeneity stems from therapeutic interventions, including CPAP therapy, upper airway surgery, and renal denervation, all of which may directly influence both oxygenation metrics and ABPM outcomes. Because treatment adherence and efficacy were inconsistently reported, it remains unclear whether hypoxemia parameters function as independent predictors of BP changes or partly reflect treatment-related effects. Although associations between nocturnal hypoxemia and circadian BP patterns were observed in both treated and untreated populations, causal inference remains limited. Future studies with standardized reporting of treatment response and adherence are needed to better define the independent predictive value of these markers.

This systematic review has important strengths, including a comprehensive search across multiple databases without restrictions on language or publication date, which minimizes selection bias and maximizes coverage of the available evidence. Nevertheless, some limitations should be acknowledged. The included studies showed methodological variability, with differences in diagnostic criteria and outcome definitions. In addition, several studies had small sample sizes and heterogeneous populations, which may limit comparability and reduce the generalizability of the findings. Publication bias could not be formally assessed through funnel plot inspection or statistical tests (e.g., Egger’s test), as quantitative pooling was not performed due to clinical and methodological heterogeneity among studies described. To address heterogeneity, sensitivity analysis was conducted through a stratified qualitative synthesis rather than quantitative pooling, providing a structured exploration of variability across studies. Evidence regarding HB remains limited, as only one eligible study evaluated this metric in relation to ABPM outcomes; therefore, the results should be interpreted as exploratory and future studies are needed to clarify its potential incremental value beyond traditional hypoxemia indices such as ODI or T90%. Collectively, these limitations should be considered when interpreting the results and highlight the need for future studies with greater methodological standardization and larger representative samples.

## Conclusion

This systematic review examined the association between blood pressure outcomes in studies using ambulatory blood pressure monitoring and indices of nocturnal hypoxemia, including the oxygen desaturation index, mean and minimum SpO_2_, T90%, and hypoxic burden. Associations across studies were heterogeneous, reflecting differences in how these indices capture the severity and duration of hypoxemia, as well as variations in study design and adjustment for confounding factors. Among the evaluated indices, ODI3% showed the most consistent association with increased blood pressure, whereas minimum SpO_2_, mean SpO_2_, and T90% demonstrated more variable findings. Hypoxic burden was assessed in only one study, suggesting a potential role in blood pressure reduction after CPAP; however, the available evidence remains preliminary. Further studies using standardized methodologies are needed to clarify the association between different hypoxemia indices with blood pressure outcomes.

## Data Availability

No new datasets were generated during the current study. All data analyzed in this systematic review were extracted from previously published studies, which are cited in the article.

## Declarations

### Clinical trial

There is no clinical trial registration number because this is not a clinical trial.
